# A phase 2a study investigating the effects of ritlecitinib on brainstem auditory evoked potentials and intraepidermal nerve fiber histology in adults with alopecia areata

**DOI:** 10.1002/prp2.1204

**Published:** 2024-07-05

**Authors:** Samira Anderson, Guido Cavaletti, Linda J. Hood, Michael Polydefkis, David N. Herrmann, Gary Rance, Brett King, Amy J. McMichael, Maryanne M. Senna, Brian S. Kim, Lynne Napatalung, Robert Wolk, Samuel H. Zwillich, Gregor Schaefer, Yankun Gong, Melanie Sisson, Holly B. Posner

**Affiliations:** ^1^ Department of Hearing and Speech Sciences University of Maryland College Park Maryland USA; ^2^ Experimental Neurology Unit, School of Medicine and Surgery University of Milano‐Bicocca Monza Italy; ^3^ Department of Hearing and Speech Sciences Vanderbilt University Medical Center Nashville Tennessee USA; ^4^ Department of Neurology Johns Hopkins University School of Medicine Baltimore Maryland USA; ^5^ Department of Neurology University of Rochester Rochester New York USA; ^6^ Department of Audiology and Speech Pathology The University of Melbourne Carlton Victoria Australia; ^7^ Department of Dermatology Yale University School of Medicine New Haven Connecticut USA; ^8^ Department of Dermatology Wake Forest School of Medicine Winston‐Salem North Carolina USA; ^9^ Department of Dermatology Lahey Hospital and Medical Center Burlington Massachusetts USA; ^10^ Harvard Medical School Boston Massachusetts USA; ^11^ Icahn School of Medicine at Mount Sinai New York New York USA; ^12^ Pfizer Inc New York New York USA; ^13^ Mount Sinai Hospital New York New York USA; ^14^ Pfizer Inc Groton Connecticut USA; ^15^ Pfizer Pharma GmbH Berlin Germany

**Keywords:** alopecia areata, audiological, axonal dystrophy, BAEP, brainstem auditory evoked potentials, intraepidermal nerve fibers, neurological, ritlecitinib, safety

## Abstract

Reversible axonal swelling and brainstem auditory evoked potential (BAEP) changes were observed in standard chronic (9‐month) toxicology studies in dogs treated with ritlecitinib, an oral Janus kinase 3/tyrosine kinase expressed in hepatocellular carcinoma family kinase inhibitor, at exposures higher than the approved 50‐mg human dose. To evaluate the clinical relevance of the dog toxicity finding, this phase 2a, double‐blind study assessed BAEP changes and intraepidermal nerve fiber (IENF) histology in adults with alopecia areata treated with ritlecitinib. Patients were randomized to receive oral ritlecitinib 50 mg once daily (QD) with a 4‐week loading dose of 200 mg QD or placebo for 9 months (placebo‐controlled phase); they then entered the active‐therapy extension and received ritlecitinib 50 mg QD (with a 4‐week loading dose of 200 mg in patients switching from placebo). Among the 71 patients, no notable mean differences in change from baseline (CFB) in Waves I–V interwave latency (primary outcome) or Wave V amplitude on BAEP at a stimulus intensity of 80 dB nHL were observed in the ritlecitinib or placebo group at Month 9, with no notable differences in interwave latency or Wave V amplitude between groups. The CFB in mean or median IENF density and in percentage of IENFs with axonal swellings was minimal and similar between groups at Month 9. Ritlecitinib treatment was also not associated with an imbalanced incidence of neurological and audiological adverse events. These results provide evidence that the BAEP and axonal swelling finding in dogs are not clinically relevant in humans.

## Graphical abstract

Results of this placebo‐controlled study, which assessed the neurological and neuroaudiological effects of ritlecitinib in adults with alopecia areata, provide evidence that the brainstem auditory evoked potential (BAEP) changes and axonal swelling finding in standard chronic toxicology studies in dogs are not clinically relevant in humans.

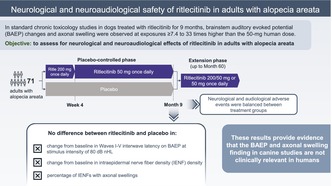

**Abbreviations**
AA: alopecia areata
AE: adverse event
AT: alopecia totalis
AU: alopecia universalis
BAEP: brainstem auditory evoked potential
BMX: bone marrow tyrosine kinase on chromosome X
BTK: Bruton's tyrosine kinase
CFB: change from baseline
EEG: electroencephalogram
IENF: intraepidermal nerve fiber
IENFD: intraepidermal nerve fiber density
IL‐15: interleukin 15
ITK: IL‐2–inducible T‐cell kinase
JAK: Janus kinase
LSM: least‐squares mean
PGI‐C: Patient Global Impression of Change
QD: once daily
SAE: serious adverse event
SALT: Severity of Alopecia Tool
TEC: tyrosine kinase expressed in hepatocellular carcinoma
TXK: resting lymphocyte kinase

## 1 INTRODUCTION

Alopecia areata (AA) is an autoimmune disease that has underlying immuno‐inflammatory pathogenesis and is characterized by nonscarring hair loss ranging from small bald patches to complete loss of scalp, face, and/or body hair.^1^ Both children and adults may be affected by AA, which has an estimated global prevalence of 2%.^2^ AA has an unpredictable disease course and may result in chronic and extensive hair loss,^3^, ^4^ which has been shown to have a widespread negative psychosocial impact on patients with AA.^5^, ^6^, ^7^, ^8^, ^9^, ^10^, ^11^, ^12^, ^13^

The underlying immuno‐inflammatory pathogenesis of AA involves collapse of the immune privilege of the hair follicle followed by recognition of hair follicle autoantigens by T‐cell receptors on cytotoxic T cells.^14^, ^15^, ^16^ Interferon γ production by T cells induces interleukin 15 (IL‐15) production and initiates a feed‐forward loop mediated by Janus kinase (JAK) signaling that further contributes to loss of immune privilege at the hair follicle and hair loss.^17^, ^18^ Downstream signaling from the T‐cell receptors involves members of the tyrosine kinase expressed in hepatocellular carcinoma (TEC) kinase family (including IL‐2–inducible T‐cell kinase [ITK]) and may also have a role in the autoimmune process of AA.^19^, ^20^

Two therapies are currently approved for the treatment of severe AA. Baricitinib, a JAK1/2 inhibitor,^21^ is approved in the United States, Japan, EU, China, and several other countries for adult patients with severe AA. Ritlecitinib, an oral, selective dual inhibitor of JAK3 and all five members of the TEC family kinases (TEC, ITK, Bruton's tyrosine kinase [BTK], bone marrow tyrosine kinase on chromosome X [BMX], and resting lymphocyte kinase [TXK]), is approved for adolescent (12–17 years of age) and adult patients with severe AA in the United States, Japan, EU, China, and several other countries. The kinases targeted are mainly expressed in the hematopoietic compartment, which include all immune cells.^22^, ^23^ The unique mechanism of action of ritlecitinib is believed to target a narrow spectrum of cytokines, which are pathogenic in AA, such as IL‐15 and IL‐2, while sparing JAK3 independent signaling. Inhibition of some members of the TEC kinase family may also confer additional benefit by dampening activation and cytolytic activity of T cells.^24^, ^25^, ^26^

In placebo‐controlled, phase 2a and 2b/3 clinical trials, ritlecitinib demonstrated efficacy and an acceptable safety profile in patients with AA.^27^, ^28^ The treatment regimen consisting of a loading dose of 200 mg once daily (QD) for the initial 4 weeks followed by 50 mg QD, is the highest dose regimen studied in patients with AA. The pharmacokinetic profile of ritlecitinib is characterized by rapid absorption and elimination, with approximately dose‐proportional exposures up to 200 mg.^29^, ^30^ Ritlecitinib is primarily metabolized by multiple glutathione S‐transferases and cytochrome P450 enzymes, with no single route contributing >25% of total metabolism.^29^, ^30^

Standard preclinical studies are required by regulatory agencies during the drug development process. In chronic (9‐month) toxicology studies in beagle dogs, reversible axonal dystrophy was observed in the central nervous system (cerebellum) at exposures ≥7.4× the 50‐mg human ritlecitinib dose (calculated based on the average of the unbound area under the curve) and in the central nervous system (superior olivary nucleus, spinal cord, and lateral lemniscus) and peripheral nervous system (branches of the vagus nerve and/or Auerbach's and Meissner's plexuses) at exposures ≥14× the 50‐mg human dose (data on file). The axonal dystrophy consisted of axonal swellings that were not associated with neuronal or axonal loss, inflammation, demyelination, or structural alterations in synapses.^31^ Brainstem auditory evoked potential (BAEP) assessments were used to determine the functional effects of axonal dystrophy in the brainstem auditory pathway. BAEP morphology of the later waves (Waves IV and V) was altered when auditory stimuli were delivered at lower intensity levels (i.e., absent or diminished amplitude) in two of 14 dogs at exposures 33× the human dose of 50 mg. There were no statistically significant increases in absolute latency (time interval between stimulus onset and appearance of a particular BAEP peak) or central transmission time (interpeak latency between Waves I and V). This BAEP finding is consistent with a centrally, not peripherally, mediated deficit in the auditory system of dogs. BAEP changes were completely reversible after a 6‐month recovery period. While axonal dystrophy was reversible at all exposure levels, at high systemic exposures (33× the human dose of 50 mg), it was considered adverse because it was associated with abnormal BAEP and because, in humans, axonal neuropathy has been associated with disordered central auditory processing/abnormal speech perception.^32^

This phase 2a, placebo‐controlled study was designed to investigate the clinical relevance in humans of the axonal dystrophy finding in dogs. This was done by assessing BAEP and intraepidermal nerve fiber (IENF) histology in humans receiving ritlecitinib. Additionally, other measures of safety and efficacy were assessed.

## 2 MATERIALS AND METHODS

### 2.1 Patient population

This phase 2a study (ClinicalTrials.gov, NCT04517864) enrolled adults aged 18–50 years at 27 sites across Australia, Canada, Poland, and the United States. Patients had a diagnosis of AA with ≥25% scalp hair loss as measured by the Severity of Alopecia Tool (SALT) (including alopecia totalis [AT; complete scalp hair loss] and alopecia universalis [AU; complete loss of scalp, facial, and body hair]). SALT is an instrument used to measure the amount of scalp hair loss, a key feature of AA, with scores ranging from 0 (no scalp hair loss) to 100 (complete scalp hair loss).^33^ Participants were required to have normal baseline hearing, BAEP, and neurological examination (one‐sided, stable ulnar, or carpal tunnel neuropathy was allowed). Exclusion criteria included hearing loss or disease that could affect hearing (including disorders associated with progressive hearing loss), history of clinically significant central or peripheral neurological disease, or first‐degree family history of hereditary neuropathy, active or chronic infection, elevated glycated hemoglobin, and previous use of a systemic JAK inhibitor.

### 2.2 Study design

**FIGURE 1:**
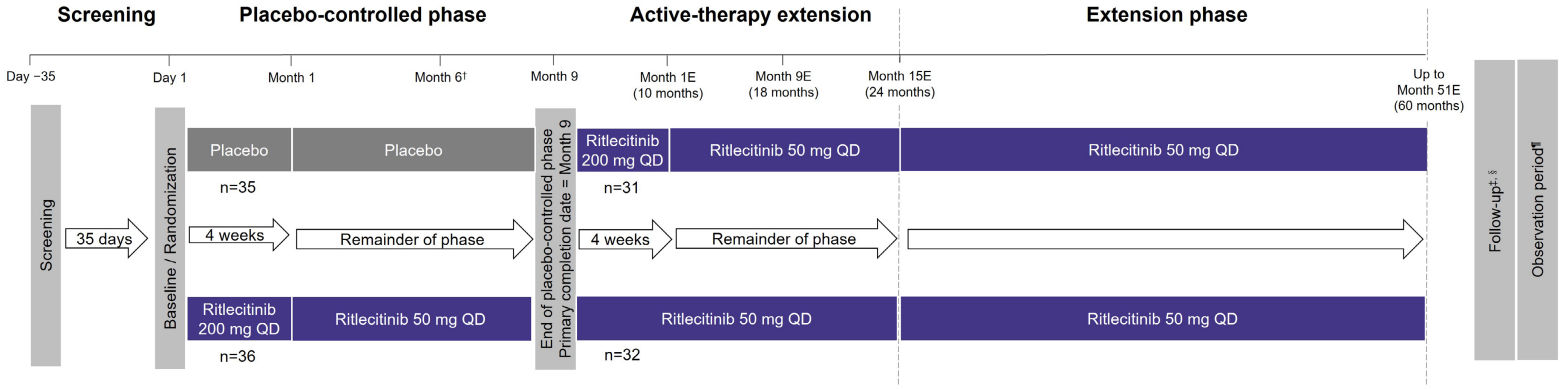
Study design. E, extension; QD, once daily; SALT, Severity of Alopecia Tool. ^†^Any patient with a baseline overall SALT score ≤75 was given the option to enter the active‐therapy extension if the overall SALT score had increased from baseline by ≥25 points at Month 6. Two patients from the placebo group with SALT score increase from baseline by ≥25 points entered the active‐therapy extension at Month 6. ^‡^After completion of the active‐therapy extension if not continuing to the extension phase or discontinuing study intervention, a follow‐up period of four weeks will occur. Patients in countries where ritlecitinib is not commercially available at the time of their Month 24 visit will have the opportunity to enter the extension phase, of variable length for individual patients for a maximum of 36 months or until availability of commercial product in their country or until the study is terminated in that country, whichever occurs first. ^§^In the extension phase, after completion or discontinuation of study intervention, a follow‐up period of four weeks will occur. ^¶^If study intervention is permanently discontinued, the patient will be asked to remain in the study after the follow‐up visit for the observation period without study intervention and continue to comply with study visit schedules for approximately two years or until study end, whichever occurs first. If a patient discontinues due to neurological or audiological event at any time during the study, a follow‐up period of six months will occur.

This was a double‐blind, parallel‐group, placebo‐controlled study. Patients were randomized 1:1 to receive either ritlecitinib 50 mg QD (after a loading dose of 200 mg QD for the initial four weeks) or placebo for nine months (Figure 1). At Month 9, patients entered the active‐therapy extension during which they received ritlecitinib 50 mg QD (with an initial 4‐week loading dose of 200 mg in patients switching from placebo). When the last patient entered this phase, the sponsor study team was unblinded to individual patient treatment assignment during the placebo‐controlled phase, while the investigators, site staff, and patients remained blinded. Patients who complete the active‐therapy extension to Month 24 have the option to continue in this phase until Month 60 or when commercial ritlecitinib is available in in their country.

At Month 6, any patient with a baseline SALT score of ≤75 (≤75% scalp hair loss) had the option to enter the active‐therapy extension if their overall SALT score at Month 6 had increased (worsened) from baseline by ≥25 points. All safety data collected until the last participant completed the placebo‐controlled phase (Month 9) or discontinued from the study are reported. BAEP and IENF results, as well as efficacy data, are reported from the placebo‐controlled phase.

The protocol was reviewed and approved by the institutional review boards or ethics committees of the participating institutions. The study was conducted in accordance with the International Ethical Guidelines for Biomedical Research Involving Human Subjects (Council for International Organizations of Medical Sciences 2002), International Council of Harmonisation Guideline for Good Clinical Practice, and the Declaration of Helsinki. Written informed consent was obtained from each patient or the patient's legal representative.

### 2.3 Outcomes

#### 2.3.1 BAEP assessments

**FIGURE 2:**
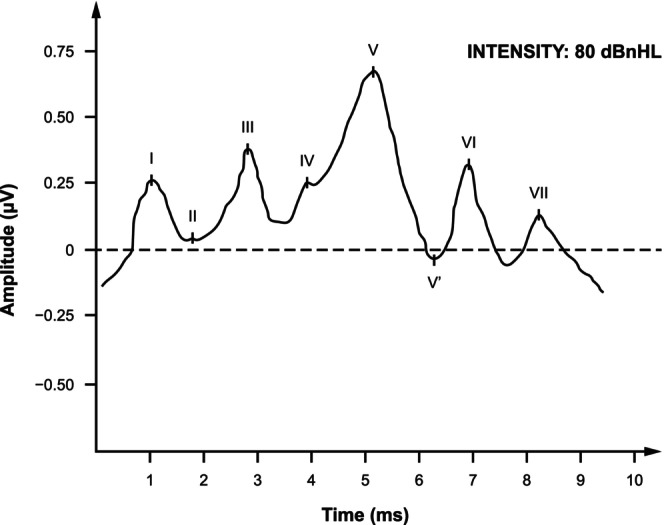
Normal BAEP waveform. BAEP, brainstem auditory evoked potential. Reprinted from Deshpande S, Houston L, Keith R. Hearing testing, auditory brainstem response (ABR). In: Kountakis SE, ed. *Encyclopedia of Otolaryngology, Head and Neck Surgery*. Berlin, Heidelberg: Springer; 2013:1151–1158. © 2013, Springer‐Verlag Berlin Heidelberg.

The primary outcome (safety) was change from baseline (CFB) in Waves I–V interwave latency on BAEP at a stimulus intensity of 80 dB nHL at Month 9 (Figure 2). Using an 80 dB nHL stimulus level, normative mean (SD) interwave latency for Waves I–V at 30 clicks/s was 4.0 (0.21) ms.^34^ Secondary safety outcomes included CFB in Waves I–V interwave latency at a stimulus intensity of 80 dB nHL at Month 6, CFB in peak‐to‐peak amplitude of Wave V to Wave V′ on BAEP at a stimulus intensity of 80 dB nHL at Months 6 and 9, and absence of Wave V at stimulus intensities ranging from 80 to 40 dB nHL at Months 6 and 9.

#### 2.3.2 IENF assessments

Secondary safety outcomes included CFB in IENF density (IENFD) and CFB in axonal swellings at Month 9 assessed in skin punch biopsies of the lateral ankle. The distal, lateral leg (10 cm above the ankle) was used for skin punch biopsies because this is a standard location for measuring IENF histology and the distal portions of long axons are more susceptible to axonopathy. In addition, the region is innervated by a sub‐branch of the sciatic nerve, in which axonal swelling was observed in the 9‐month dog studies.

IENFD was measured by counting the number of axon fibers that independently crossed the dermal‐epidermal barrier (basement membrane) and extended into the epidermis by at least one keratinocyte. Secondary branches and axon fiber fragments within the epidermis were not counted. The length of epidermis in each section was measured, and the linear IENFD was reported as number of IENFs/mm. Four 50‐μM‐thick skin sections were quantified, and the average was used as the final value.

For assessments of axonal swellings, any IENF with single or multiple swellings was counted as a single event, that is, a single axon with axonal swellings. For each patient, data were reported as the percentage of IENFs with swellings.^35^ Histological analyses for IENFD and axonal swelling were performed by an independent central reader at Johns Hopkins University laboratory who was blinded to all treatment assignments.

#### 2.3.3 Adverse events and serious adverse events

Adverse events (AEs), serious AEs (SAEs), and AEs leading to discontinuation were recorded, along with the incidence of clinically significant abnormalities in vital signs and clinical laboratory values. A central laboratory was used for safety laboratory tests. Four independent safety adjudication committees evaluated AEs of special interest, including (1) opportunistic infections, (2) cardiovascular events, (3) neurological (including audiological) events, and (4) malignancies. The Neuro Safety Events Adjudication Committee, a blinded external adjudication committee, comprised neurology experts as well as subspecialists in neuroaudiology, who provided targeted assessments of neurological and audiological events. Potential events of interest were identified during the routine monitoring of patient study records. In addition, the treatment‐emergent AE listings were searched for prespecified preferred terms in the Medical Dictionary for Regulatory Activities System Organ Classes: ear and labyrinth disorders, eye disorders, nervous system disorders, and psychiatric disorders. Events were adjudicated by the external Neuro Safety Events Adjudication Committee to determine whether they met criteria for a neurosafety event of interest.

#### 2.3.4 Efficacy

Efficacy outcomes were assessed as secondary endpoints at Month 9 and other time points and included CFB in SALT score and the Patient Global Impression of Change (PGI‐C) score, defined as “greatly improved” or “moderately improved” AA.

### 2.4 Audiological and neurological evaluations

Audiological evaluations (including audiological history, otoscopic examination, pure tone audiometry [air and bone conduction], speech audiometry, and immittance audiometry) were performed by an audiologist at screening and at Months 6 and 9. Clinically significant changes were recorded as AEs (regardless of whether there had been a complaint of hearing issues). Audiological and BAEP evaluations were performed within 7 days of each other (on the same day, if possible) with audiological assessment first. Manuals and study guides were used to standardize conventional audiological and BAEP evaluation parameters across study sites. Each conventional audiology and BAEP assessment was reviewed by one of three central readers who were expert neuroaudiologists. The central reader confirmed at each visit that assessments were performed per study parameters and that locally read BAEP waves were labeled appropriately and at their peak so that latency and amplitude data were accurate. Only results confirmed by the central reader as accurately interpreted were used for analysis. A neurological examination was performed at screening and at Months 6 and 9 by a qualified (board certified or equivalent) neurologist and included a general neurological evaluation and a neuropathy assessment. Audiological, BAEP, and neurological data were collected and hosted by WorldCare Clinical.

### 2.5 Statistical analysis

The planned sample size of 30 patients per group was based on the primary endpoint. Assuming an SD of 0.2 ms based on published findings for Waves I–V interwave latency on BAEP (range, 0.1–0.3 ms)^34^ and assuming the SD of CFB is similar to the SD of actual scores, the half‐width of 95% CIs for the group would be 0.07 ms for 30 patients per group.

CFB in Waves I–V interwave latency on BAEP at a stimulus intensity of 80 dB nHL at Month 9 was analyzed using a linear mixed‐effects model with baseline, treatment group, visit, and treatment group by visit interaction as fixed effects with unstructured covariance matrix assumption. For patients who switched to the active‐therapy extension at Month 6, only their data through Month 6 were included in the analysis of the placebo‐controlled period. Descriptive statistics for continuous variables were used for CFB in axonal swellings and IENFD in skin punch biopsies at Month 9. For the absence of Wave V on BAEP at stimulus intensities ranging from 80 to 40 dB nHL at Months 6 and 9, data were summarized descriptively using number and percentage of patients by treatment group at each intensity level.

CFB in SALT score during the placebo‐controlled phase was analyzed using a linear mixed‐effects model with baseline, treatment group, visit, and treatment group by visit interaction as fixed effects with unstructured covariance matrix assumption. For PGI‐C response during the placebo‐controlled phase, number and percentage with 95% CIs (based on the Clopper–Pearson method) by treatment group and treatment difference with 95% CIs (based on the Chan and Zhang exact method^36^) are presented.

## 3 RESULTS

### 3.1 Patients

**FIGURE 3:**
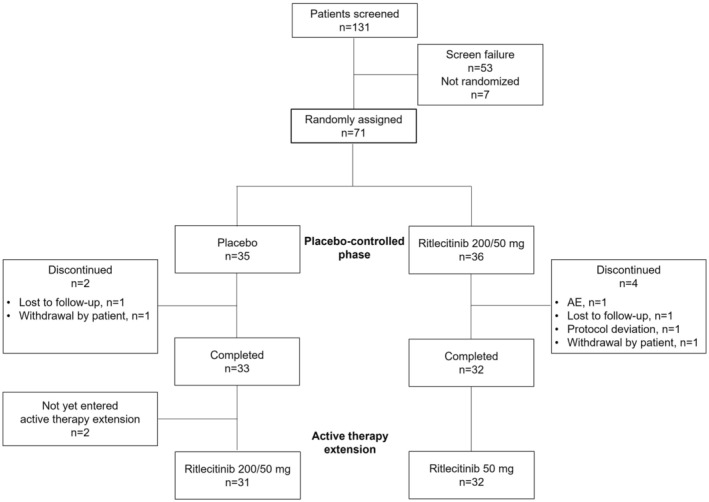
Patient disposition. AE, adverse event.

A total of 71 patients were randomized to receive either ritlecitinib 200/50 mg (*n* = 36) or matching placebo (*n* = 35) (Figure 3). The mean (SD) age of patients was 34.7 (9.2) years and 70.4% were female (Table 1). Overall, 76.1% of patients were White, 15.5% were Black or African American, and 4.2% were Asian; 11.3% were Hispanic/Latino. The median duration of AA since primary diagnosis and current episode of hair loss due to AA were 8.9 and 3.0 years, respectively. Per study protocol, patients with AT/AU were defined as having SALT scores of 100. The mean (SD) SALT score for non‐AT/AU patients was 56.9 (27.6) at baseline (Table 1). Among all patients, each of whom had a normal neurological examination (a single upper extremity neuropathy not withstanding), the mean (SD) IENFD was 10.6 (3.9) at baseline, which was consistent with published normal ranges. Mean (SD) percentage of IENFs with axonal swelling was 1.8% (2.3%) at baseline; there are no generally accepted normative data for baseline percentage of IENFs with axonal swelling.

**TABLE 1:** Baseline demographic and clinical characteristics.

	Placebo (*n* = 35)	Ritlecitinib 200/50 mg QD (*n* = 36)	Total (*N* = 71)
Age, years			
18–25, *n* (%)	6 (17.1)	6 (16.7)	12 (16.9)
26–35, *n* (%)	13 (37.1)	14 (38.9)	27 (38.0)
36–45, *n* (%)	13 (37.1)	10 (27.8)	23 (32.4)
46–50, *n* (%)	3 (8.6)	6 (16.7)	9 (12.7)
Mean (SD)	34.2 (9.0)	35.1 (9.6)	34.7 (9.2)
Sex, *n* (%)			
Female	25 (71.4)	25 (69.4)	50 (70.4)
Male	10 (28.6)	11 (30.6)	21 (29.6)
Race, *n* (%)			
White	28 (80.0)	26 (72.2)	54 (76.1)
Black or African American	4 (11.4)	7 (19.4)	11 (15.5)
Asian	0	3 (8.3)	3 (4.2)
Multiracial	2 (5.7)	0	2 (2.8)
Not reported	1 (2.9)	0	1 (1.4)
Ethnicity, *n* (%)			
Hispanic or Latino	5 (14.3)	3 (8.3)	8 (11.3)
Baseline percentage of nerve fibers with axonal swelling			
*n*	35	35	70
Mean (SD)	1.8 (2.1)	1.8 (2.5)	1.8 (2.3)
Baseline IENFD (/mm)			
*n*	35	35	70
Mean (SD)	11.0 (4.0)	10.2 (3.8)	10.6 (3.9)
Baseline SALT scores for non‐AT/AU patients			
*n*	23	27	50
Mean (SD)	53.7 (24.2)	59.6 (30.3)	56.9 (27.6)

*Note*: *n* values in column heads are the patient populations in each treatment group; *n* values in rows are the number of patients with valid data.

Abbreviations: AT, alopecia totalis; AU, alopecia universalis; IENFD, intraepidermal nerve fiber density; SALT, Severity of Alopecia Tool.

Six patients discontinued during the placebo‐controlled phase, four in the ritlecitinib 200/50‐mg group (one due to an AE [Takayasu arteritis] that was not considered to be treatment related) and two in the placebo group (Figure 3). A total of 63 patients entered the active‐therapy extension at Month 9, of whom 32 were initially randomized to ritlecitinib and continued the maintenance 50‐mg dose (extension ritlecitinib 50‐mg group) and 31 were initially randomized to placebo and then switched to ritlecitinib 200/50 mg (extension ritlecitinib 200/50‐mg group). Two patients from the placebo group entered the active‐therapy extension at Month 6 due to SALT score increase (worsening) of ≥25 points at Month 6. The median duration of exposure during the placebo‐controlled phase and active‐therapy extension was 9.0 and 1.3 months, respectively, for the patients randomized to ritlecitinib and 8.9 and 1.4 months for the patients randomized to placebo and switched to ritlecitinib.

### 3.2 BAEP assessments

There was no notable mean CFB in Waves I–V interwave latency on BAEP at a stimulus intensity of 80 dB nHL within the ritlecitinib 200/50‐mg or the placebo groups on the right or left side at Month 9 (Table 2). No notable differences in Waves I–V interwave latency between the two groups on either side were observed. The mean Waves I–V interwave latency values remained within the range of published normative data (mean [SD]: 4.0 [0.21] ms)^34^ used to define normality for inclusion of patients into the study. Only one patient, who was in the placebo group, had lengthened Waves I–V interwave latency beyond two SDs of the published mean (4.0 ms)^34^ at Month 9, before transition to ritlecitinib treatment. Review by a panel of neuroaudiology experts concluded that the BAEP results for this patient did not suggest any neurological safety concerns.

**TABLE 2:** Primary and secondary outcomes.

	Placebo (*n* = 35)	Ritlecitinib 200/50 mg QD (*n* = 36)	Difference from placebo
Primary endpoint			
LSM (SE) [95% CI] CFB in Waves I–V interwave latency (ms) on BAEP at 80 dB at Month 9			
*n*	32	31	‐
Right side	−0.010 (0.027) [−0.063 to 0.043]	0.011 (0.027) [−0.043 to 0.065]	0.021 (0.038) [−0.056 to 0.097]
Left side	0.022 (0.021) [−0.020 to 0.065]	0.031 (0.022) [−0.012 to 0.075]	0.009 (0.031) [−0.052 to 0.070]
Secondary endpoints			
LSM (SE) [95% CI] CFB in Waves I–V interwave latency (ms) on BAEP at 80 dB nHL at Month 6			
*n*	34	34	‐
Right side	−0.024 (0.021) [−0.065 to 0.017]	−0.030 (0.021) [−0.072 to 0.011]	−0.006 (0.030) [−0.065 to 0.053]
Left side	−0.020 (0.016) [−0.053 to 0.012]	0.021 (0.016) [−0.011 to 0.054]	0.042 (0.024) [−0.005 to 0.088]
CFB in IENFD in skin punch biopsies at Month 9			
*n*	33	32	‐
Mean (SD)	−0.2 (2.7)	−0.4 (3.9)	‐
CFB in IENFD in skin punch biopsies normalized by age and sex at end of placebo‐controlled phase^a^			
*n*	33	32	
Mean (SD)	−4.1 (43.1)	−6.0 (63.0)	
CFB in percentage of nerve fibers with axonal swelling in skin punch biopsies at end of placebo‐controlled phase^a^			
*n*	33	32	‐
Mean (SD)	−0.2 (2.4)	0.6 (2.4)	‐
LSM (SE) [95% CI] CFB in peak‐to‐peak amplitude of Wave V to Wave V′ (μV) on BAEP at 80 dB nHL			
At Month 6, *n*	34	34	‐
Right side	−0.017 (0.016) [−0.048 to 0.015]	−0.031 (0.016) [−0.063 to 0.000]	−0.015 (0.022) [−0.059 to 0.030]
Left side	−0.019 (0.017) [−0.053 to 0.015]	−0.047 (0.017) [−0.082 to −0.013]	−0.028 (0.024) [−0.076 to 0.020]
At Month 9, *n*	32	31	‐
Right side	0.008 (0.016) [−0.025 to 0.041]	−0.051 (0.017) [−0.085 to −0.018]	−0.060 (0.024) [−0.107 to −0.012]
Left side	−0.049 (0.018) [−0.085 to −0.012]	−0.045 (0.019) [−0.082 to −0.008]	0.003 (0.026) [−0.049 to 0.056]

*Note*: *n* values in column heads are the patient populations in each treatment group; *n* values in rows are the number of patients with valid data.

Abbreviations: BAEP, brainstem auditory evoked potential; CFB, change from baseline; LSM, least‐squares mean; IENFD, intraepidermal nerve fiber density; QD, once daily.

^a^ For the two patients who entered the active‐therapy extension phase at Month 6, end of placebo‐controlled phase refers to Month 6.

At Month 6, there was also no notable mean CFB in Waves I–V interwave latency on BAEP at a stimulus intensity of 80 dB nHL within the ritlecitinib 200/50‐mg or placebo groups on either side at Month 6 (Table 2). No notable differences in Waves I–V interwave latency between the two groups on either side were observed at Month 6.

At Months 6 and 9, mean CFB and mean percent CFB in amplitude of Wave V on BAEP at a stimulus intensity of 80 dB nHL on the right and left sides were minimal in both treatment groups (Table 2). There were no notable differences in the mean change in amplitude or mean percent change in amplitude of Wave V from baseline between the two groups. No patient had an absence of Wave V on BAEP at any intensity level on the left side up to Month 9. All patients had Wave V present on BAEP at stimulus intensities ranging from 80 to 40 dB nHL on the right side up to Month 9, except for one patient. At Month 9, one patient in the ritlecitinib 200/50‐mg group had an absence of Wave V on BAEP at a stimulus intensity of 40 dB nHL on the right side. The event was unilateral and showed fluctuations in the presence or absence of Wave V at various intensities on repeated assessments starting at Month 6. Hearing sensitivity remained normal from screening through Month 9. Review by a panel of neuroaudiology experts concluded that there was no evidence of neural transmission abnormalities in the auditory nerve or auditory brainstem and that the likely explanation for the absence of Wave V was that the evoked response amplitude was too small for it to be identified within the electroencephalogram (EEG). On follow‐up evaluation after Month 9, this patient had normal BAEP waveforms at all intensities and normal hearing in both ears.

### 3.3 IENF assessments

The IENFD mean (SD) and median (Q1, Q3) values for both ritlecitinib and placebo were consistent with published normal ranges both at baseline and Month 9. There were no meaningful changes in mean or median IENFD in ritlecitinib and placebo groups (Table 2). IENFD results were further analyzed by normalizing the data using the fifth percentile reference values (provided by the laboratory that performed the IENFD measurements) based on age and sex. Mean (SD) CFB in IEFND normalized by age and sex was −6.0 (63.0) in the ritlecitinib group and −4.1 (43.1) in the placebo group.

**FIGURE 4:**
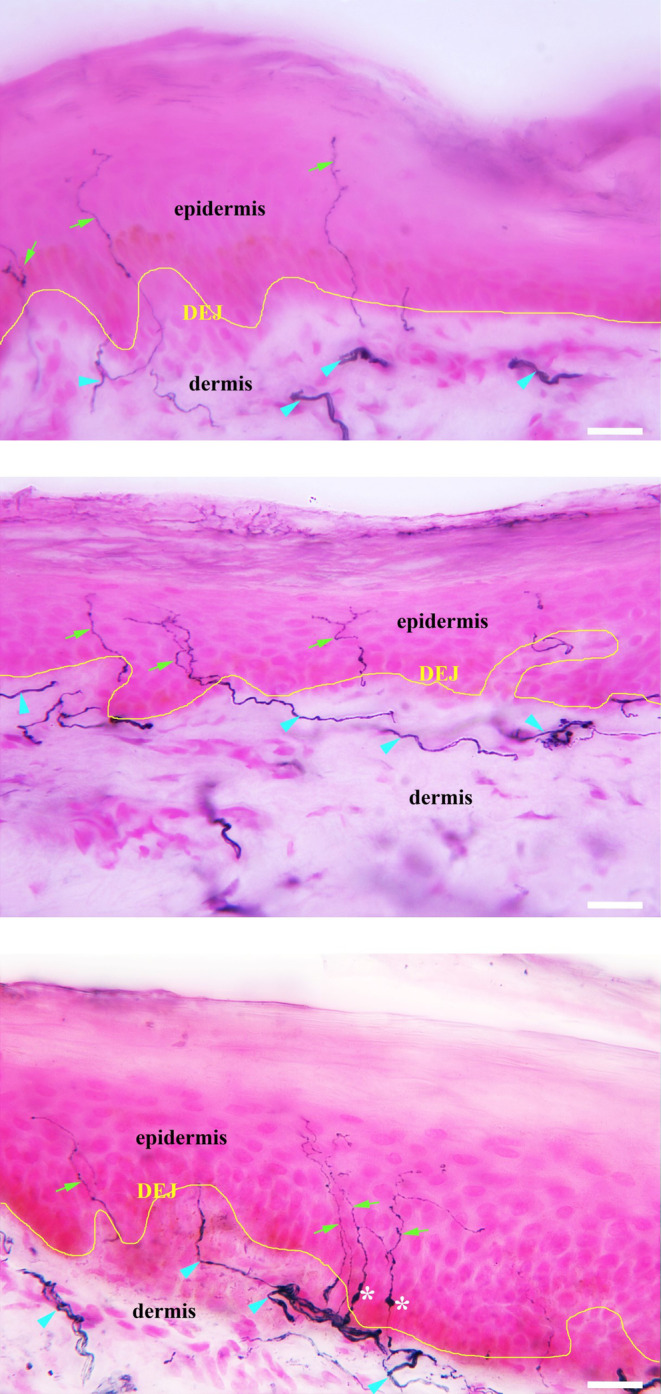
Baseline images of IENF histology. DEJ, dermal‐epidermal junction; IENF, intraepidermal nerve fiber; SENF, subepidermal nerve fiber. Green arrows indicate IENFs. Blue arrowheads indicate SENFs. White asterisks indicate nerve swellings. The white bar indicates 60 microns.

The CFB in percentage of IENFs with axonal swellings was minimal and similar at the end of the placebo‐controlled phase between the ritlecitinib and placebo groups (Table 2). Baseline images of IENF histology are shown in Figure 4.

### 3.4 AEs and SAEs

During the placebo‐controlled phase, 78 AEs were reported in 29 patients (80.6%) in the ritlecitinib 200/50‐mg group and 57 AEs were reported in 22 patients (62.9%) in the placebo group (Table 3). In the active‐therapy extension phase, three patients who switched from placebo to ritlecitinib and three who continued ritlecitinib experienced AEs. Two patients had SAEs (neither considered treatment related by the investigator): One patient in the placebo group had a humerus fracture during intense exercise but continued the study, and one patient in the extension ritlecitinib 50‐mg group had Takayasu arteritis (seven years after a right subclavian artery occlusion cleared by angioplasty) and was discontinued from the study. One patient in the ritlecitinib 200/50‐mg group discontinued the study drug due to an AE of prostatitis (not considered treatment related by the investigator) but continued the study visits without receiving ritlecitinib. No deaths were reported.

**TABLE 3:** Summary of AEs.

	Placebo‐controlled phase^a^		Active‐therapy extension	
	Placebo (*n* = 35)	Ritlecitinib 200/50 mg QD (*n* = 36)	Extension Ritlecitinib 200/50 mg QD (*n* = 31)	Extension Ritlecitinib 50 mg QD (*n* = 32)
Total no. of AEs	57	78	3	3
Patients with ≥1 AE, *n* (%)	22 (62.9)	29 (80.6)	3 (9.7)	3 (9.4)
Leading to withdrawal from study^b^	0	0	0	1 (3.1)
Leading to withdrawal from drug but not from study^c^	0	1 (2.8)	0	0
Leading to temporary drug discontinuation	1 (2.9)	6 (16.7)	0	0
Patients with ≥1 SAE, *n* (%)	1 (2.9)	0	0	1 (3.1)
Patients with severe AEs, *n* (%)	2 (5.7)	0	0	0
AEs occurring in ≥5% of patients				
COVID‐19	3 (8.6)	3 (8.3)	0	0
Nasopharyngitis	1 (2.9)	2 (5.6)	1 (3.2)	1 (3.1)
Headache	1 (2.9)	4 (11.1)	0	0
Acne	2 (5.7)	2 (5.6)	0	0
Acne pustular	0	4 (11.1)	0	0
Dizziness	1 (2.9)	3 (8.3)	0	0
Hypoesthesia	3 (8.6)	1 (2.8)	0	0
Nausea	3 (8.6)	1 (2.8)	0	0
Vomiting	1 (2.9)	3 (8.3)	0	0
Upper respiratory tract infection	1 (2.9)	2 (5.6)	0	1 (3.1)
Treatment‐related AEs occurring in ≥5% of patients				
Acne pustular	0	4 (11.1)	0	0

*Note*: Includes all data collected since the first dose of study drug. Except for the no. of AEs, patients are counted only once per treatment in each row.

Abbreviations: AE, adverse event; QD, once daily; SAE, serious adverse event.

^a^ For patients who switch to the active‐therapy extension phase at Month 6, only their data through Month 6 were included in the analysis.

^b^ Patients who had an AE record indicating that the AE caused the patient to be discontinued from the study.

^c^ Patients who had an AE record indicating that action taken with study treatment was drug withdrawn but AE did not cause the patient to be discontinued from study.

Seven patients (9.9%) had study drug interruptions due to AEs (six in the ritlecitinib 200/50‐mg group and one in the placebo group); three of the drug interruptions in patients in the ritlecitinib 200/50‐mg group were considered by investigators to be treatment‐related AEs. The most frequently reported AEs (by preferred term; >5%) in the total population were COVID‐19 (*n* = 6 [8.5%]) nasopharyngitis, headache (*n* = 5 [7.0%] each), acne, acne pustular, dizziness, hypoesthesia, nausea, vomiting, and upper respiratory tract infection (*n* = 4 [5.6%] each). Of these, nasopharyngitis, headache, acne pustular, vomiting, upper respiratory tract infection, and dizziness were reported in higher proportions of patients in the ritlecitinib 200/50‐mg group than the placebo group. There was one opportunistic infection (multidermatomal herpes zoster) in the ritlecitinib 200/50‐mg group; the event was mild in severity and not considered treatment related. All treatment‐related AEs were mild (16 events) or moderate (6 events) in severity. AEs in nine patients (four in the ritlecitinib 200/50‐mg group and five in the placebo group) were adjudicated as meeting criteria for neurological event of interest terms.

In the ritlecitinib group, neurological events of interest included asthenia (*n* = 1) and paresthesia and/or dysesthesia (*n* = 3). In the placebo group, neurological events of interest included somnolence (*n* = 1), dizziness (not vertigo or presyncope; *n* = 1), and paresthesia and/or dysesthesia (*n* = 3). No events met the criteria for event of interest term peripheral neuropathy. Additionally, no events met the adjudication criteria for an audiological event of interest term (including sensorineural hearing loss or central hearing disorder). No clinically meaningful CFB in mean hematology, lipids, liver, and chemistry laboratory parameters were observed, and there were no clinically meaningful changes in vital signs.

### 3.5 Efficacy (secondary endpoints)

SALT scores decreased from baseline through Month 9, with numerically greater least‐squares mean (LSM) CFB in SALT score in the ritlecitinib 200/50‐mg group than in the placebo group (−38.2 [95% CI, −47.5 to −28.9] vs. −6.8 [95% CI, −16.1 to 2.4]; difference, −31.3 [95% CI, −44.4 to −18.2]) (Table 4). A larger proportion of patients in ritlecitinib 200/50‐mg group were PGI‐C responders (defined as greatly improved or moderately improved) than in the placebo group at Months 3, 6, and 9 (55.6% vs. 17.1%, 58.3% vs. 28.6%, and 52.8% vs. 17.1%, respectively). The 95% CI for the difference between groups excluded 0 at Months 3, 6, and 9 for both CFB SALT scores and PGI‐C responders.

**TABLE 4:** Exploratory efficacy outcomes.

	Placebo (*n* = 35)	Ritlecitinib 200/50 mg QD (*n* = 36)	Difference from placebo
LSM (SE) [95% CI] CFB in SALT score			
At Month 3	−2.7 (3.4) [−9.39 to −4.1]	−23.0 (3.4) [−29.7 to −16.2]	−20.3 (4.8) [−29.8 to −10.8]
At Month 6	−5.1 (4.7) [−14.4 to 4.2]	−35.2 (4.7) [−44.6 to −25.8]	−30.1 (6.6) [−43.3 to −16.8]
At Month 9	−6.8 (4.6) [−16.1 to 2.4]	−38.2 (4.7) [−47.5 to −28.9]	−31.3 (6.6) [−44.4 to −18.2]
PGI‐C response^a^			
At Month 3, *n* (%)	6 (17.1)	20 (55.6)	‐
	‐	‐	38.4 (10.4) [13.8 to 58.0]
At Month 6, *n* (%)	10 (28.6)	21 (58.3)	
	‐	‐	29.8 (11.2) [5.2 to 51.1]
At Month 9, *n* (%)	6 (17.1)	19 (52.8)	‐
	‐	‐	35.6 (10.5) [11.5 to 55.4]

Abbreviations: CFB, change from baseline; LSM, least‐squares mean; PGI‐C, Patient Global Impression of Change; QD, once daily; SALT, Severity of Alopecia Tool.

^a^ PGI‐C response was defined as a PGI‐C score of moderately improved or greatly improved.

## 4 DISCUSSION

In this phase 2a, placebo‐controlled study undertaken to assess the clinical relevance of the axonal dystrophy finding from the standard 9‐month dog toxicity studies, there were no notable mean CFB within either the ritlecitinib or placebo groups, or between the two groups, in any of the BAEP or IENF parameters studied at Month 9. The results of the current study, with the absence of any effect of ritlecitinib on IENF and BAEP and no concerning findings regarding neurosafety AEs, support the neurological and audiological safety of ritlecitinib. This conclusion is also supported by the observation that the axonal dystrophy (swelling) finding in the standard dog toxicity studies was species specific (i.e., it was not observed in standard rodent studies of up to six months' duration [data on file]), adverse effects in dogs were observed at ritlecitinib exposures exceeding those of human therapeutic doses for AA (≥7.4 to 33× the 50‐mg human ritlecitinib dose), and BAEP changes were reversible and only observed at the highest exposures (33× the 50‐mg human dose). Hence, the non‐clinical findings in dogs are not clinically relevant in humans.

Because the BAEP finding was considered a central auditory effect in the dog toxicity studies, BAEP Waves I–V interwave latency at 80 dB nHL was chosen as the primary endpoint for this study. Generally, the interwave latency at high intensities is the standard BAEP measurement used to assess the neural integrity of the brainstem auditory pathway and for diagnosis of brainstem effect in humans.^37^ Additionally, this measurement has a high test–retest reliability in humans.^38^ There was no notable mean CFB in Waves I–V interwave latency at 80 dB nHL on BAEP in either the ritlecitinib or placebo groups up to Month 9 in patients with AA, with no notable differences in mean Waves I–V interwave latency between the two groups. The mean Waves I–V interwave latency values remained within the published normative data.^34^ There is no universally accepted minimal increase in Waves I–V interwave latency that is considered clinically meaningful; however, audiologists often use >2 SDs (0.42 ms) beyond the published mean (4.0 ms)^34^ as a starting point for increased surveillance. One patient in the placebo group had lengthened Waves I–V interwave latency beyond two SDs of the published mean^34^ at Month 9; however, a panel of expert neuroaudiologists concluded that BAEP results for this patient did not suggest any neurological safety concerns.

Although Waves I–V interwave latency on BAEP using high‐intensity stimulus is the standard measurement for assessing auditory brainstem integrity in humans, the secondary endpoints measuring Wave V amplitude and presence/absence of Wave V at lower intensities were also evaluated to reflect the observation in dogs of altered BAEP morphology of the later waves (absence or diminished amplitude of Waves IV and V) at lower‐intensity levels. These endpoints are not standardly used to assess the neural integrity of the human brainstem. There were no notable differences in the change in amplitude or percent change in amplitude of Wave V from baseline in the ritlecitinib or placebo groups or between the two groups. One patient in the ritlecitinib group had unilateral absence of Wave V on BAEP at a stimulus intensity of 40 dB nHL at Month 9. However, a panel of expert neuroaudiologists found no evidence of neural transmission abnormalities in the auditory nerve or auditory brainstem, and the panel suggested as a possible explanation that the evoked response amplitude was too small to be identified in the EEG. On the final follow‐up evaluation, while on ritlecitinib at Month 24, this patient had normal BAEP waveforms at all intensities bilaterally.

Skin biopsies at the lateral distal leg within the distal territory of the sciatic nerve, the longest nerve in the human body, allowed direct assessments of both morphological features of nerve endings (such as axonal swellings) and IENFD in an area corresponding to one affected in dogs and in a location prone to show the effects of toxins that cause neuropathy.^39^, ^40^, ^41^ IENFD has reference values stratified by age and sex^40^ and is extensively used in the clinic, whereas reference values for axonal swellings are less well‐characterized. Small changes observed in IENFs in the absence of a consistent association of these changes with clinical symptoms are not considered clinically meaningful. CFB to Month 9 in mean IENFD or in percentage of IENFs with axonal swellings was minimal and similar between the ritlecitinib 200/50‐mg and placebo groups.

Overall, ritlecitinib was generally safe and well‐tolerated; most AEs were mild or moderate in severity, and no treatment‐related SAEs were reported. The number of patients with AEs meeting the criteria as neurological events of interest was balanced between treatment groups in the placebo‐controlled phase. There were no events that met the criteria for audiological events of interest, including sensorineural hearing loss and central hearing disorder. The safety and tolerability of ritlecitinib in this study were consistent with those in the phase 2A ALLEGRO trial (NCT02974868), the ALLEGRO phase 2b/3 trial (NCT03732807), the ongoing phase 3 open‐label, long‐term ALLEGRO‐LT (NCT04006457) study, and the integrated safety analysis of pooled data from the aforementioned ALLEGRO studies and the present study.^27^, ^28^, ^42^

As a result of the chronic toxicology results in dogs, additional neurological and audiological safety evaluations and event adjudication were conducted proactively across the ALLEGRO clinical trial program. AEs adjudicated by an independent external committee to meet the criteria for an audiological event of interest reflected the outcomes of protocol‐specified audiological testing (even in the absence of spontaneously reported AEs related to hearing) and spontaneously reported AEs related to hearing. The integrated safety analysis of data pooled from four studies in the ALLEGRO program included 881 placebo‐controlled patients and 1294 patients in the any‐ritlecitinib cohort (patients who received ≥1 dose of ritlecitinib in any of the four studies; 2092 total patient‐years), of whom 1228 patients received ritlecitinib 50 mg with or without a 200‐mg loading dose (ritlecitinib 50‐mg cohort; 1814 patient‐years).^42^ In this integrated safety analysis, no central hearing disorder AEs or serious neurological AEs were reported, and no evidence of neurotoxicity with ritlecitinib was demonstrated.^42^ Furthermore, neurological events of interest did not demonstrate characteristics of acute or chronic cumulative injury to axons in the central or peripheral nervous system.^42^

In the present study, improvements in SALT and PGI‐C scores were also consistent with previous studies of ritlecitinib in patients with AA.^27^, ^28^

This study has some limitations. By design, patients with certain neurological and audiological conditions were excluded; however, the purpose of this neuroaudiological and neurological safety study of ritlecitinib was to investigate the clinical relevance in humans of the axonal dystrophy finding in dogs, and the exclusion criteria were chosen to avoid possible confounding of results because of underlying neurological and audiological conditions. Furthermore, the other ALLEGRO clinical trial program studies, which also did not show any evidence of neurotoxicity with ritlecitinib, had less restrictive exclusion criteria than the present study in terms of pre‐existing neurological and audiological conditions. The present study was limited to patients aged 18–50 years. Patients >50 years of age were excluded to avoid potential confounding of study results because of the increased risk of neuropathy and audiological issues related to older age. Younger patients were excluded as a population generally considered “vulnerable” to participate in research studies, but the findings and conclusions from this study are considered applicable to younger patients who receive ritlecitinib because the nervous system corresponding to the relevant regions where axonal changes were observed in dogs (including auditory pathways and peripheral nervous system) is fully developed in humans by the age of six years.^43^, ^44^, ^45^, ^46^, ^47^, ^48^, ^49^, ^50^, ^51^ Finally, although there was some diversity in the patient population, approximately three‐quarters of patients were White.

## 5 CONCLUSIONS

Results of the current study support the neuroaudiological and neurological safety of ritlecitinib, with no notable changes on evaluation of BAEP and IENFs and no concerning findings related to neurosafety AEs. The current study, along with the integrated safety analysis of over 1200 ritlecitinib‐treated patients representing 2092 patient‐years of exposure,^42^ provide further evidence that the axonal dystrophy finding in dogs is not clinically relevant in humans.

## AUTHOR CONTRIBUTIONS

S. Anderson, R. Wolk, S.H. Zwillich, M. Sisson, and H.B. Posner were involved in the conceptualization and design of this study. Y. Gong performed the formal analysis of the study. S. Anderson, L.J. Hood, and G. Rance were involved in central oversight of BAEP measurements. M. Polydefkis performed IENF histology analyses. G. Schaefer, M. Sisson, and H.B. Posner wrote the original manuscript draft. S. Anderson, G. Cavaletti, L.J. Hood, M. Polydefkis, D.H. Herrmann, G. Rance, B. King, A.J. McMichael, M.M. Senna, B.S. Kim, L. Napatalung, R. Wolk, S.H. Zwillich, G. Schaefer, Y. Gong, M. Sission, and H.B. Posner contributed to the critical review and editing of the manuscript. R. Wolk, S.H. Zwillich, M. Sisson, and H.B. Posner provided supervision. H.B. Posner provided project administration.

## FUNDING INFORMATION

This study was funded by Pfizer.

## CONFLICT OF INTEREST STATEMENT

SA is a neuroaudiological consultant to Pfizer. GC has served on advisory boards and/or been a consultant and/or clinical trial investigator for Algo Therapeutics, Augustine Therapeutics, Egetis Therapeutics, Eli Lilly, Hoba Therapeuticx, Nanosilical, Pfizer, Plasma Protein Therapeutic Association Europe, Toray Industries, UCB Biopharma, and Voluntis Digital Therapeutics. LJH is a neuroaudiological consultant to Pfizer. She received research grants from the National Institutes of Health and Akouos. MP has served on advisory boards and/or been a consultant for Vertex, Alnylam, AstraZeneca, Intellia, and Teva. DNH has served on advisory boards and/or been a consultant for Acceleron Pharma, Regenacy, Pfizer, Sarepta, Neurogene, Applied Therapeutics, DTx Pharma, NMD Pharma, Passage Bio, Swan Bio, Faze Medicines, GLG, Slingshot Insights, and Guidepoint Global. GR is a neuroaudiological consultant to Pfizer. BK has received honoraria and/or consultation fees from AbbVie, AltruBio, Almirall, AnaptysBio, Arena Pharmaceuticals, Bioniz Therapeutics, Bristol Myers Squibb, Concert Pharmaceuticals, Equillium, Horizon Therapeutics, Eli Lilly, Incyte, Janssen Pharmaceuticals, LEO Pharma, Otsuka/Visterra, Pfizer, Regeneron, Sanofi Genzyme, TWi Biotechnology, and Viela Bio. He previously served on speaker bureaus for AbbVie, Incyte, Eli Lilly, Pfizer, Regeneron, and Sanofi Genzyme. AJM has received grants/research funding from Concert, Procter and Gamble, and Incyte and consulting fees from Eli Lilly, Janssen, Pfizer, Arcutis, Almirall, AbbVie, Galderma, Bristol Myers Squibb, Sanofi Genzyme, UCB, Procter and Gamble, Revian, Johnson & Johnson, L'Oreal, and Nutrafol. MMS has served on advisory boards and/or been a consultant and/or clinical trial investigator for Arena Pharmaceuticals, Concert Pharmaceuticals, Eli Lilly, LEO Pharma, and Pfizer. She is a speaker for Eli Lilly and Pfizer. BSK is founder of Klirna Biotech; he has served as a consultant for 23andMe, ABRAX Japan, AbbVie, Almirall, Amagma Therapeutics, Arcutis Biotherapeutics, Arena Pharmaceuticals, argenx, AstraZeneca, Bellus Health, Blueprint Medicines, Boehringer Ingelheim, Bristol Myers Squibb, Cara Therapeutics, Clexio Biosciences, Cymabay Therapeutics, Daewoong Pharmaceutical, Eli Lilly, Escient Pharmaceuticals, Evommune, FIDE, Galderma, Genentech, GlaxoSmithKline, Granular Therapeutics, IQVIA, Incyte Corporation, Innovaderm Research, Janssen Research & Development, Kiniksa, LEO Pharma, Locus Biosciences, Maruho, Medicxi, Menlo Therapeutics, Novartis, OM Pharma, Pfizer, Recens Medical, Regeneron Pharmaceuticals, Sanofi and Genzyme US Companies, Septerna, Shaperon, Third Harmonic, Vial, and WebMD; he has stock in ABRAX Japan, KliRNA Biotech, Locus Biosciences, and Recens Medical; he holds a patent for the use of JAK1 inhibitors for chronic pruritus. LN, RW, GS, YG, MS, and HBP are employees of and may hold stock or stock options in Pfizer. SHZ was an employee of Pfizer at the time of the study and manuscript preparation and may hold stock or stock options in Pfizer.

## ETHICS STATEMENT

The protocol was reviewed and approved by the institutional review boards or ethics committees of the participating institutions. The study was conducted in accordance with the International Ethical Guidelines for Biomedical Research Involving Human Subjects (Council for International Organizations of Medical Sciences 2002), International Council of Harmonisation Guideline for Good Clinical Practice, and the Declaration of Helsinki.

## PATIENT CONSENT STATEMENT

Written informed consent was obtained from each patient or the patient's legal representative.

## Data Availability

Upon request, and subject to review, Pfizer will provide the data that support the findings of this study. Subject to certain criteria, conditions, and exceptions, Pfizer may also provide access to the related individual de‐identified patient data. See https://www.pfizer.com/science/clinical‐trials/trial‐data‐and‐results for more information.
